# Stillbirth registration and perceptions of infant death, 1900–60: the Scottish case in national context^1^

**DOI:** 10.1111/j.1468-0289.2009.00478.x

**Published:** 2009-08

**Authors:** GAYLE DAVIS

**Affiliations:** University of Edinburgh

## Abstract

The history of vital registration has attracted substantial attention from both social historians and historical demographers. While much of that research has touched upon issues of fertility and mortality, the contentious issue of the stillborn child—which falls somewhere between the two—has been largely neglected. Although civil birth and death registration was introduced to Scotland in 1855, stillbirth registration did not begin until 1939. Using a range of legal, medical, and statistical evidence, this article explores the history of stillbirth registration in Scotland from a social history perspective. It outlines the problems associated with lack of stillbirth registration, the processes that eventually led to registration of the stillborn child, and the wider significance of that registration.

The foetal mortality rate has come to be regarded as a valuable historical health status indicator.^2^ Thus, the omission of stillbirths for almost the first century of British civil registration has arguably constituted a major difficulty for both doctors and statisticians. Stillbirths only became a part of the vital statistics of England and Wales through the Births and Deaths Registration Act of 1926;^3^ and it took Scotland over a decade to follow suit, through the Registration of Still-Births (Scotland) Act 1938.^4^ However, this latter legislation included an important and innovative addition—that a statement of the cause or probable cause of death was required from the doctor or midwife in attendance. Thus, although the stillbirth problem was a national issue, the Scottish case is in some respects particularly worthy of study. The statistics produced by the Registrar General for Scotland following stillbirth registration, and his analyses of that data, quickly became an invaluable resource for those wishing to gain greater insight into the problem of stillbirth in Britain and its implications for public health.

The history of civil registration has received fairly detailed attention from scholars in recent years.^5^ The beginnings of national death registration and the compilation of cause-of-death statistics have been seen, for example, to offer potentially rich insights into the complex interaction between the state, the public, and the legal and medical professions.^6^ While a number of such studies have focused specifically upon infant mortality, concerning themselves with the death of babies less than a year old but live-born, very few have incorporated stillbirths.^7^ This relative absence of stillbirth within the historiography can at least partly be explained by the dearth of data before registration, and the questionable reliability of that data after it. The early history of vital registration in Britain, as Higgs makes clear, reflects the prioritization of legal rather than public health concerns, stillbirths not being registered because the stillborn child was not considered to have a legal existence.^8^ The lack of scholarly interest in stillbirths could therefore also be said to reflect the historically impoverished legal status of the stillborn child.

In addition, previous research into British civil registration has centred mainly on England. Scotland, although the subject of sophisticated demographic research,^9^ and with distinctive medical and statistical traditions, has attracted little attention from a social history perspective.^10^ Yet the Scottish experience was by no means parochial, since the registration methods in England and Scotland often progressed through an iterative process of experiment in one system being followed by ‘improvements’ in the other. An emphasis here on Scottish material is also justifiable because records concerning the history of registration have been far better preserved in Scotland than in England, and hence throw light on practices in both countries.

Using a range of medical, statistical, and government files, including hitherto unused records which have been made available by the Registrar General for Scotland, this article explores the social history of stillbirth registration. It outlines the problems associated with lack of stillbirth registration; and then explores the motives—social, medical, and statistical—behind the eventual registration of stillbirths. Finally, it considers the wider significance of that registration. While stillborn children were considered unworthy of permanent record because they lacked a legal existence, their numbers and significance were difficult to estimate; but registration of stillbirth focused widespread medical and statistical attention on the stillborn for the first time as a public health issue, and thereby helped to transform perceptions of the newborn and stillborn child. Thus it will be argued that Armstrong's contention that the Registrar General's creation of a distinct mortality rate for infants both reflected an emerging social awareness of infant mortality, and created social, statistical, and medical recognition of the infant as a separate and important entity, can be extended to foetal mortality.^11^

## I

As the *Scotsman* noted in 1855, Scotland finally had, ‘after several futile attempts’, obtained an act to provide better registration of births, deaths, and marriages, an Act ‘long and much wanted’.^12^ Yet the Registration (Scotland) Act of 1854 revealed ‘a manifest omission’ in the non-registration of stillborn children. Neither the ‘birth’ nor ‘death’ of stillborn children was required to be registered, whereas any child born alive—no matter how brief its survival—must have both its birth and death registered. As the newspaper asked: ‘Most assuredly they are born, and why should they not be registered?’^13^ Indeed, as soon as compulsory civil registration came into force in Scotland, the question of whether or not to register stillbirths alongside live births became a contentious issue. Registration examiner reports, registrar queries, and the press all regularly noted confusion and concern over the omission of stillborn children from the vital statistics of Scotland.^14^

This omission appears to have been mainly because the system was designed to record the natality of legal rather than biological persons. As the medical jurist John Glaister noted, the Registration Acts took ‘no interest’ in stillbirths because ‘the still-born child, as the old legal phraseology puts it, never was “a reasonable creature in being, and under the King's peace”, [and thus] the State can have no concern over an infant which never had a legal existence’.^15^ The stillborn were thus treated as if they had never existed, and registered as neither a birth nor a death.

Nevertheless, several difficulties in relation to this non-registration were raised in the early examiners' reports. For example, it was noted that, in almost all cases of registration of infant death in Dundee, the child was reported to the registrar to have been stillborn. However, on closer enquiry, due to the sheer number of stillbirths reported in this region, it was discovered that the child had in fact ‘lived for a shorter or a longer space of time’.^16^ Another examiner noted that ‘sextons and undertakers may call any body which they bury (in a small coffin) a still born child, and so escape the penalty following on the neglect of sending information to the Registrar’.^17^ Even more disturbingly, the registrar of the district of Partick ‘had reason to believe that in his district children though born alive, but dying through the neglect or instrumentality of their parents immediately after birth, [were] frequently given out by them as having been “still born” ’, and accordingly not registered in either birth or death registers.^18^ Thus, as another examiner enquired: ‘How many cases of “Still Birth” are in point of fact, accidental death? How many are, in the eye of the moral law, infanticide? How many are the result of the social misery, starvation, or depravity of the mother?’^19^

On a more statistical note, the Perth examiner lamented that it was ‘a great pity Stillborn children were excluded from Registration’, this omission operating against ‘a true return of the number of Births being given’, and an aspect of fertility which would ‘form a valuable branch in medical statistics’.^20^ Indeed, several Registrars General for Scotland complained along similar lines. Stair Agnew's 1877 Annual Report noted the absence of stillbirth returns, ‘a circumstance which statisticians and physiologists cannot but regret’. He continued: ‘Those disposed to question the utility of such returns may be reminded that, without such information as they would furnish, the mortality due to the process of childbearing cannot be ascertained year by year with the desirable precision’.^21^

A survey of the General Register Office for Scotland (hereafter GROS) and press correspondence in the early decades of the twentieth century reveals stillbirth to be a continuing problem for local registrars and examiners. There could be conflicting statements between parents and the attending doctor, or midwife and doctor, that a child had been live- or stillborn, although the general rule in the registration process was that any doctor present at the birth ‘must naturally be the best authority on the question whether the child was born alive or not’.^22^ This was despite medical disputes over the ‘diagnosis’ of stillbirth at this time. The *Edinburgh Medical Journal* reported a case which, ‘although possibly very rare’, should ‘make one careful’ in regarding the traditional test of floating the lungs in water as proof of live birth,^23^ and ‘even still more careful’ in estimating the value of Breslau's stomach–bowel test.^24^ Another writer was ‘obliged to confess’ to a coroner that, had he not had the ‘indisputable evidence’ of five different people on oath that the child lived five hours, he ‘should be compelled to swear that the child was stillborn, and had never breathed’.^25^ Indeed, as one doctor noted, ‘[i]t was common to hear a medical man say: “I was not sure whether it was still-born or dead” ’.^26^

There was a more significant concern that some stillbirths were in fact cases of infanticide, and the ‘stillbirth’ label merely a convenient classification to avoid suspicion. Through the late nineteenth and early twentieth centuries, there was widespread social concern over the issue of infanticide.^27^ John Glaister, the doyen of Scottish medical jurisprudence at this time, devoted a chapter of his medico-legal bible *Medical jurisprudence and toxicology* to the subject.^28^ As he explained, in any such charge within Scotland, the law started from the presumption that the child had been born dead until proof of foul play was adduced, the *onus probandi* thus falling upon the prosecutor. The child then had to be proven to have been both viable and completely separate from its mother before the act of destruction had taken place, making the infanticide charge very difficult to prove.^29^

A related concern was the issue of disposal of stillborn children.^30^ Before stillbirth registration, stillborn babies could be buried without the production to the gravedigger of any forms of medical certificate or declaration statutorily required in Scotland.^31^ The Scottish Board of Health advised that there was no legal obligation on doctors to certify a child stillborn in either Scotland or England, and that it was not the general practice among doctors to do so. They added that it ‘frequently happen[ed] that bodies of still-born children [were] buried in places other than cemeteries or Burial Grounds’.^32^ It was, however, possible for a cemetery authority to lay down, as one of the conditions governing burials in their cemetery, that the body of a stillborn child would not be buried there unless a certificate by a doctor or certified midwife was furnished to the cemetery superintendent, and practice seems to have varied widely on this matter.^33^

Medical journals thus regularly drew attention to the ‘remarkably unsatisfactory state of the law on this matter’.^34^ One author noted: ‘The ease with which the body of a child alleged to be stillborn can be got rid of is a disgrace to our Statute Book’.^35^ The use of the word ‘alleged’ is particularly interesting here. As an earlier *British Medical Journal* article had noted, there was evidence that children who died within 24 hours of birth ‘were constantly received and buried as “stillborn” ’ due to the fact that such a form of burial was ‘cheap and expeditious, and [did] not involve a funeral service’.^36^ The article added that this had the ‘additional advantage of secrecy, which would naturally commend it to those who desire[d] for some reason to withhold the fact of the birth from the public view’. Thus, in the present state of the law, nothing stood between ‘the quiet putting away of a child which [was] only “stillborn” because it ha[d] not been allowed to live but the caution of cemetery authorities in accepting declarations from persons who [we]re neither medical men nor coroners’.^37^

It was thus a fairly frequent worry that children who were live-born but died soon afterwards might be claimed to have been stillborn in order to save the reputation of an unmarried mother or to justify a burial ‘on the cheap’. For example, in 1927 a registrar recorded that a Glasgow undertaker had behaved in a ‘highly irregular fashion’^38^ by falsifying burial certificates of stillborn children and burying two in a single coffin. This behaviour was, the procurator-fiscal stated, ‘grossly irregular’, and caused ‘a tremendous amount of trouble’, presumably motivated by the fact that, by altering the certificates and burying two bodies in one box, he saved the 5 s. burial fee and the price of one box in each interment.^39^ An agent speaking for the undertaker explained that he had been in business for only two years, and that he understood that such a procedure was quite usual.^40^ However, the procurator-fiscal stated that methods of disposing of stillborn children, or children who died shortly after birth, had been causing a great deal of difficulty to the police and the authorities generally. The local registrar reported subsequently that a conviction had been obtained in this case, and a ‘satisfactory penalty imposed’. The undertaker was fined £25, with the alternative of 30 days' imprisonment, the sheriff expressing the hope that it would act as a warning to undertakers that this sort of thing could not go on. As the registrar concluded: ‘The penalty and the publicity given this case will in a large measure shake up the undertaking fraternity who carry on business [similarly]’.^41^

It was not clear in some cases whether the undertaker was defrauding the parents by charging them individually for collective burials when only one had taken place, or conniving with them to reduce burial costs. Indeed, despite moral and legal concerns, parents seem generally to have consented—for either economic or emotional reasons—when a doctor offered to remove the body of the stillborn child to a maternity hospital or university for pathological purposes. Such instances were occasionally reported to both the Scottish Board of Health and the procurators-fiscal amid concerns that these bodies were subsequently disposed of in the hospital furnace, apparently in contravention of the terms of the 1902 Cremation Act.^42^ Ultimately, however, no proceedings were taken against hospitals involved in this practice.^43^

Such concerns were also common to England, and were one of the factors that led to the Births and Deaths Registration Act of 1926. As a *Lancet* article summed up, in relation to two medical men in Sheffield accused of wilfully giving a false certificate of stillbirth, the difficulties relating to stillbirth were acute. It was extremely difficult ‘to determine the precise moment at which life cease[d]’, despite the fact that ‘important issues might hang upon the hour named by the medical man testifying to a death’, such as the devolution of property, or a possible charge of manslaughter or murder.^44^ In the Sheffield case, there was said to be no evidence of any motive except for possibly a ‘saving of fees’ to the relatives. However, as the *British Medical Journal* noted, the fact remained that it was ‘perfectly easy not only to kill intentionally and with the basest motives a perfectly healthy child during or immediately after birth, without running the risk of a charge of murder, but [also] to secure its interment without a medical certificate and without inquiry’.^45^ Opportunities throughout Britain for immorality, crime, and monetary gain were thus plentiful in a system that did not require any notification of the existence of a stillborn child. Furthermore, the vital statistics of Scotland, England, and Wales were being compromised by the exclusion of stillborn children.

Growing concern in Britain over this issue must be set within the context of acute early twentieth-century anxiety over ‘national efficiency’, infant mortality, and the declining birth rate. As the medical officer of health for Glasgow, A. K. Chalmers, stated in 1904: ‘In any review of the changes which have taken place in the causes of death during the last thirty years, few things stand more in need of investigation than the persistently high range of the mortality among infants during the first year of life’.^46^ He noted that, in order to make better progress in this field, ‘a more intimate acquaintance with the conditions preceding infant deaths was required’.^47^ This would require the Registration Act to be amended so that early knowledge of births might be made available, since almost one-third of infant deaths occurred during the first four weeks of life, and yet in Scotland 21 days and in England 42 could elapse before a birth was registered. Similar concerns were raised in the Scottish press, with even greater emphasis during the First World War. The *Glasgow Herald* devoted a leading article to the ‘disquieting prospect’ indicated in the Returns of the Registrars General of Scotland and England regarding the steady decline of the British birth rate, which had ‘become portentous’ since the outbreak of war. In Scotland alone, 9,730 fewer births were recorded in 1915 than in the preceding year, with a simultaneous rise in the infantile mortality rate. Scotland was visibly ‘burning the candle at both ends’, producing fewer children and losing more of its ‘precious asset’ than ever before, augmented by the Great War which was making ‘murderous inroads on the young [and] virile’.^48^

As a pre-war step towards combating this wastage of infant life, the Early Notification of Births Bill was introduced in 1907. It provided for notification to the local health authority of every birth within 36 hours of its occurrence, such notice to be given by the father if resident in the house at the time, or, failing that, by any person in attendance on the mother. This included the birth of every child that had reached a gestation period of 28 weeks, even if stillborn. This legislation was said to be unpopular with the medical men and midwives on whom it imposed a new obligation of reporting promptly all births that they had attended, with no remuneration offered for their service, and on penalty of ‘suffering the indignity of being summoned to the police-court’ and fined. Although the Act was applicable to the whole of Great Britain, it was amended in Committee of the House of Commons into an adoptive measure, applicable only at the request of a local authority, and subject to that authority's satisfying the Local Government Board that they could administer it satisfactorily.^49^

Notification of births was immediately adopted in both Glasgow and Edinburgh.^50^ It did not provide an absolute check on birth registration, and there were discrepancies between the number of stillbirths registered at cemeteries and the number actually notified.^51^ However, an analysis of findings after the first year of its operation in Glasgow revealed that the 1907 proportion of still to live births, previously estimated at 5 per cent, was in fact barely 3 per cent, or 2.4 per cent in the case of births not attended by a doctor. It was assumed that midwives were less rigorous than doctors in notifying stillbirths, and the case of a midwife convicted in the Sheriff Court for failing to notify a stillbirth revealed that many midwives were in fact ignorant of their responsibility.^52^ As a result of this prosecution, and the placing of notices in the offices of the district registrars, the number of stillbirths notified quickly increased, and this category of births was, for the first time, recognized statistically. By 1914, more than 50 per cent, or nearly 2.5 million Scots, were under notification,^53^ although the Act had not been adopted by all local authorities, with scattered rural districts and less populous centres tending to ignore it.^54^ The Notification of Births (Extension) Act of 1915, however, made birth notification obligatory,^55^ giving local authorities a greater opportunity to study ‘the conditions which might imperil infant life’ and ‘to put at the disposal of parents such skilled advice as their circumstances demanded’.^56^

Yet it was recognized that infant mortality statistics published in different countries were not comparable at this time. The Royal Statistical Society of London appointed a Special Committee in 1911 to conduct a comparative enquiry into birth and infant death registration systems in different countries.^57^ Inquiry forms—136 in total—were sent to all the registration officers of the ‘civilized states of the world’, with 103 replies received.^58^ The survey revealed wide discrepancies between countries in the recording of infantile mortality and stillbirth, ‘a veritable jungle of legislative, official, and lexicographic divergencies of theory and practice’.^59^ They concluded that registration of stillbirths ought to be established in Britain and all other countries, and that this required a satisfactory international definition of stillbirth. Dr Reginald Dudfield concurrently published an influential paper which deplored the impossibility of international comparisons as matters stood.^60^ He argued that efficient registration would allow the examination of ‘the causes of unnecessary and preventable wastage of life’, in which case vital statistics could be regarded as ‘taking the place of laboratory experiments in social physiology and pathology’.^61^ However, sound conclusions clearly could not be obtained unless the basic data were reliable and accurate, and the methods of calculations used by different inquirers uniform, or at least strictly comparable.

In an article urging the registration of stillbirths in Britain at this time, J. W. Ballantyne, the physician to the Edinburgh Royal Maternity Hospital and a pioneer of antenatal pathology and hygiene, summarized the ‘amazing confusion’ into which the subject of stillbirth registration had fallen.^62^ The French system required birth registration within three days of birth, during which time any child who had died was registered as stillborn (*mort-né*), regardless of whether it had been born alive. This applied also to Belgium, the Netherlands, and Spain, where stillbirths included children dying between birth and registration. This categorization required a new term for children born alive but dying before declaration—*dits mort-nés*. Definitions differed widely as to the length of antenatal life required for a foetus to come within the scope of registration. Thus, in Hungary, children born dead after seven months' gestation were registered as stillborn, while in Japan only four months' gestation was required. Other systems considered the length of the foetus. In the German state of Hesse, a foetus was registered if not less than 30 centimetres long. In New York, the child had to have ‘attained sufficient development to determine sex’, thus theoretically carrying the earliest age for registration much further back in antenatal life. However, all of these systems at least included some kind of stillbirth registration, unlike Great Britain and Ireland.

## II

England moved more quickly towards stillbirth registration than Scotland, although it came as part of a broader measure relating to death certification and disposal.^63^ The Births and Deaths Registration Act of 1926 aimed mainly to provide stronger safeguards against the concealment of crime in relation to the disposal of the dead. Local authorities could arrange for the medical inspection in certain instances of the body of the deceased, and could regulate certain means of disposal. Furthermore, stillbirths were required to be registered for the first time.^64^ As the English Registrar General, Sylvanus Vivian, noted: ‘the absence of such registration has for many years been recognised as a defect in our registration system both on account of its importance as a safeguard for the protection of infant life and also on account of the value of a knowledge of the facts as to the frequency of stillbirths in the elucidation of the causes of both antenatal and post-natal mortality’.^65^ The Act came into effect in July 1927 but did not apply to Scotland or Northern Ireland. It was decided that Scotland be omitted from the Bill largely because many of the administrative provisions were ‘peculiarly English’ and thus inapplicable north of the border.^66^

Nonetheless, this and earlier bills on the subject dealt with matters of considerable relevance to Scotland, necessitating discussion north of the border.^67^ The assistant secretary of the Scottish Board of Health wrote to the Registrar General for Scotland, Dr James Crauford Dunlop, that, although many of the provisions of these bills were unsuitable for application to Scotland, there were ‘certain points’ within them, ‘notably the registration of still-births’, which appeared to the Board to be of value. Dunlop was asked for his department's observations on the English proposals and their views on securing similar powers for Scotland.^68^

Of the various ‘controversial principles’ being considered in England, Dunlop stated himself to be most against the registration of stillborn children. Although he recognized the statistical value of the information, he felt that this did not necessitate stillbirth registration. His main arguments were legal: that all events registered at this time dealt with civil or individual rights in some form, whereas for a stillborn child ‘with no separate existence, no civil rights, and not even a name’, there appeared to be little, if anything, to record permanently in a register. Thus, as he asked, ‘Why encumber either a birth register, a death register, or even a special register with useless detail?’^69^ Dunlop suggested that registration of stillbirths would be ‘distinctly objectionable in that it expose[d] to public search and extract … private and intimate detail with which the public [was] not concerned’. This Registrar General for Scotland, the only medical man to have held that office, believed the disposal of stillborn children to be ‘essentially a medical or medico-legal matter and one much more akin to the functions of a medical man than to those of a lay Registrar’. He recommended that the medical officer of health be given the duty of keeping a record of stillbirths, to provide greater medical scrutiny and to avoid publicity.^70^

However, through the 1930s, support grew for stillbirth registration in Scotland. The (Cathcart) Committee on Scottish Health Services was appointed in 1933, with a wide remit to report on health services and to recommend health policy.^71^ In 1936, it reported favourably on evidence submitted regarding the compulsory registration of stillbirths, and observed that, although stillbirths were notifiable to the medical officer of health, such information would be of greater value if it included the reputed causes of death.^72^ Dunlop's successor as Scottish Registrar General, James Gray Kyd, accepted the value of stillbirth notification but argued that it should be a national policy and should include the presumed cause of death, as in the case of children born alive.^73^ This decade also saw mounting concern over persistently high levels of maternal mortality and morbidity—as many as six maternal deaths per 1,000 live births in Scotland by the mid-1930s—into which a whole series of investigations were conducted throughout Britain.^74^ Medical authorities began to argue that all mortality connected with later pregnancy and in and around actual childbirth should be considered as one, and that one should ‘inevitably conclude that no problem in present-day medicine exceeds in importance that presented to us by deaths during child-bearing and in early infancy’.^75^

By the later 1930s, during a second wave of anxiety over national efficiency and infantile mortality, a range of medical and statistical arguments were marshalled in support of stillbirth registration in Scotland. Since the Scottish Department of Health had long desired stillbirth registration, which ‘might throw light on problems of the wastage of infant life and puerperal morbidity and mortality’, it was suggested that a Scottish Registration of Still-Births Bill might be allotted to a private member.^76^ Scotland lagged behind significantly in its efforts to save infant life. In 1936, the infant mortality rate was 82 for Scotland, as compared to 59 for England and Wales, and 31 for New Zealand.^77^ It was calculated with regret that, if the Scottish rate had been the same as the English, there would have been 2,000 fewer infant deaths in Scotland in that year alone. Also taking into account the number of stillbirths that were being revealed by notification, it was said to be ‘remarkable that this holocaust of infant life’ had ‘not aroused public opinion’.^78^ Without full knowledge of the number of stillbirths in Scotland, it was argued that this problem could only be partially examined.^79^ Moreover, unlike the death rates from many other causes, the stillbirth rate had not fallen in response to the ‘improvements in the environmental and other conditions of community life effected in past years’, and contrasted sharply with the falling rates of mortality in ‘practically every other period of life’.^80^ Statisticians also argued for the introduction of stillbirth registration to bring Scotland in line with England.^81^ On the basis of such arguments, the Secretary of State for Scotland proposed that cabinet authority should be sought for the introduction of a government bill to provide for the registration of stillbirths in Scotland.

A range of arguments was marshalled to propel the Scottish Registration of Still-Births Bill through Parliament. The Bill's promoter, Basil Neven-Spence, MP for Orkney and Shetland, argued that it was ‘somewhat painful for a Scottish Member to have to confess that Scotland, almost alone among civilised nations’, did not provide for the registration of stillbirths. While the distinction between stillbirth and death immediately after birth might ‘not seem to be a very substantial one’, the crucial issue for him was not whether a child had been born alive or dead, but the fact that it had been born at all. Neven-Spence defended the importance of vital statistics to medical research and public health administration, but did not wish to stress this unduly because it was ‘rather like handling a live hedgehog’. He appealed to the House on medical and statistical grounds, but also reminded his colleagues of the ‘mass of human suffering’ that lay behind these infantile mortality and other percentages.^82^

Parliamentary objections to the Bill included confusion as to how it might contribute to reducing maternal or infantile mortality. The ‘Red Clydeside’ group of Independent Labour MPs was particularly hostile.^83^ George Buchanan deemed stillbirth registration ‘a needless intrusion into the lives of, in many cases, poor people’, which would ‘do nothing to bring about better social conditions’, and might disclose the ‘rather alarming’ abortion statistics in Glasgow.^84^ David Kirkwood invoked the ‘decent lassie’ working in a shop or factory who had been ‘let down’ and simply desired ‘to forget this sort of thing’.^85^ She would ‘regard it as a godsend’ if the child was born dead. In such circumstances, surely it was cruel to ‘reduce this type of woman to the level of the English’, where women would be forced to register what ‘they regard[ed] as their greatest disaster, when they would like the whole thing to be buried’.

In response, members were asked ‘to consider whether the whole history of the improvement of the social conditions of this country’ had not been founded upon the Registration Acts. During the early industrial revolution, with its consequent ‘holocaust of children’ in the mines and factories, no one registered such deaths, ‘no inquests were held on them, and nobody worried about them’. It was only once the state ‘said they were human beings’, and their causes of deaths were looked into, that ‘the whole social conscience of the country was stirred’. Thus, as the Secretary of State for Scotland, Walter Elliot, argued: ‘It may well be that if attention were concentrated on them they would not be still-born, but living children’.^86^ The Bill passed without a division.

In the House of Lords, Lord Alness supported registration of stillbirths because it would harmonize the law of Scotland with that of England, and because it would constitute a valuable tool in medical research, casting a ‘flood of light’ on maternal and infantile mortality ‘and many other cognate problems as well’. He also cited humanitarian grounds, the ‘background of human suffering and sorrow’ which lay behind such statistics, and the ‘poignant tragedy of a dead child’, accentuated by the knowledge ‘that the tragedy was really preventable’.^87^

The Registration of Still-Births (Scotland) Act applied to any child which had issued forth from its mother after the twenty-eighth week of pregnancy and which did not at any time, after being completely expelled from its mother, breathe or show any other signs of life. The registrar was to take note of when and where the child was born, its sex, the name of the parents and rank or profession of the father, date and place of marriage, when and where registered, and the signature of both the informant and the registrar.^88^ Furthermore, the informant was to deliver to the registrar a certificate stating that the child was not born alive, signed by the registered medical practitioner who had attended the birth or examined the body of the child, or a certified midwife who was in attendance or examined the body in the absence of a doctor. Upon registering the stillbirth, the registrar was to give to the informant certification that he had registered the stillbirth, without fee, for the use of the undertaker or other person having charge of the burial.

In Article Three of the Regulations for Registrars in relation to stillbirth, certain modifications were made to the normal rules of death certification. These limited the period for registration to three months after the stillbirth, omitted any provision for issuing extracts from the register (except by the special permission of the Registrar General for Scotland), and stipulated that no duplicate be made of the register of stillbirths. These modifications were made because the stillbirth register was intended to be used to study the wastage of child life, and because a stillborn child had no civil status; thus the provision of a permanent record for legal or civil purposes was seen as unnecessary.^89^

## III

The Registration of Still-Births (Scotland) Act came into operation on 1 January 1939, extending the Scottish death registration process to any child born without sign of life after the twenty-eighth week of pregnancy. Essentially the Act extended the operation of the existing legislation for England and Wales to Scotland, but with one important addition—that a statement of the cause or probable cause of death was required from the doctor or midwife who certified the stillbirth. This scheduling of the causes of stillbirth was seen to be innovative and potentially highly useful to those interested in the subject of infant mortality, the Registrar General for Scotland noting that it was the first official attempt to generate such a record in Britain since in England only the fact of the stillbirth had to be registered.^90^

In November 1937, while the Scottish Registration of Still-Births Bill was being prepared, the Scottish Registrar General had written to his English counterpart, Sylvanus Vivian, about possible difficulties of implementation, asking whether cause of stillbirth had ever been considered when the bill that introduced English stillbirth registration was being drafted and, if so, why it was omitted.^91^ The response was that, despite much discussion in England and on Committees of the League of Nations, it had been deemed a ‘matter of extreme difficulty even for a registered medical practitioner to diagnose the cause of a stillbirth, and that in fact a comparative small proportion of stillbirths [were] attended by registered medical practitioners’.^92^ A League of Nations ‘Committee of Experts’, of which Vivian was a member, argued that classification by causes was impossible while the great majority of stillbirths were attended only by midwives.

At a subsequent meeting with the Department of Health for Scotland, Vivian confirmed that it might be difficult to register causation in many cases, and that Kyd would be ‘sorry if inclusion of Cause would endanger the Bill itself’.^93^ However, the Department of Health for Scotland was not impressed by such criticisms. Although most births at this time were at home rather than in hospital, and were not followed by a post-mortem examination, midwives were obliged to call a doctor in an emergency which, they argued, a stillbirth surely was. Furthermore, in Scotland the proportion of births that were attended by a doctor was higher than in England, so it was argued that many Scottish stillbirths were medically attended.^94^ Moreover, as the English Registrar General himself accepted, even if useful material was not forthcoming for some time, at least a beginning would have been made.^95^ The Department also suggested that an effect of the requirement to state cause of death would be that practitioners would learn to regard stillbirths ‘as something worthy of their full attention’, which would in itself ‘be fruitful of good results’ in time.^96^ As for remarks about the difficulty of classification, the answer was ‘first get your Causes and then see about classifying them’.^97^ In short, it was considered a defect of the English legislation that cause of stillbirth was not registered.^98^

Since the requirement to register cause of death was innovatory, there was much initial confusion because of the absence of an international stillbirth classification system. When the GROS invited the Department of Health for Scotland to provide examples of the most likely causes of stillbirth to be certified once registration came into force, the department's response was that they ‘[did] not, of course, know what [would] be the principal certified causes of still birth in Scottish experience’, and that the literature on the subject gave ‘results differing so widely according to the nature of the investigation’ that the suggestions they could give would only include ‘important and not necessarily the most important probable causes of stillbirths’.^99^ Therefore, from 1937 onwards, the GROS attempted to collect data from a selection of doctors, midwives, and registrars on which to base a cause of stillbirth classification. Internal correspondence suggests they scrutinized midwives' registers in order to frame a preliminary cause list of stillbirths.^100^ Furthermore, a few selected registrars were asked if they would spend the early months of 1939 noting all causes of stillbirth and submitting them to the Registrar General.^101^

Upon registration, there were two further major concerns. First was the feasibility of having all stillbirths medically certified. Successive annual reports of the Scottish Registrar General indicated the proportion of stillbirths where medical attendance was lacking. In the first year of registration, 3,616 of 3,832 stillbirths were registered by a medical practitioner, and a further 205 by a certified midwife.^102^ In the following year, the proportion certified by a medical practitioner was ‘rather higher’ than in the previous year, and that by a midwife ‘rather lower’.^103^ Great pains were taken to ensure that a medical practitioner certified the stillbirth wherever possible. Registrars and examiners policed this system so that all concerned knew their duty. Midwives were encouraged to call a doctor rather than certify the stillbirth alone.^104^ It was found that: ‘A tactful word to the doctors concerned or to the Matron in the case of events occurring in hospital [was] usually all that [was] necessary'.^105^ Whether midwives should be allowed to certify stillbirths, and whether certification would be more accurate if only doctors undertook this task, was a leitmotiv throughout the 1940s. Correspondence with the GRO in London indicated that the proportion of midwives' certificates in Scotland was ‘comparatively small’, amounting to 3.2 per cent in 1945 and only 1.6 per cent by 1948, although the Scottish Office stated itself to be satisfied by midwives' certificates, with few having to be returned for further information.^106^

The second problem was ill-defined or unknown causes. In the early years of the Act's operation, these categories contained the largest number of cases, amounting to a staggering 1,263 (of 3,832) cases in 1939 (33 per cent of the total number of stillbirths registered in Scotland in this year), although this figure quickly dropped to 755 (20 per cent) in 1941 and 590 (16 per cent) in 1942 while the number of stillbirths remained fairly constant.^107^ This was, similarly, a problem in other countries that compiled such statistics, such as the Netherlands. However, the Department of Health for Scotland contended early on that, ‘even if it were found that the cause was in a large proportion of cases “unknown” this, in itself would show that there was a socio-medical problem calling for scientific investigation’ and might focus attention on the subject.^108^

Throughout the 1940s, GROS correspondence reveals a number of complaints or difficulties experienced by those attempting to certify causes of stillbirth. One registrar reported: ‘Some medical men may be of opinion that the precise cause of [stillbirth] can be determined only by [post-mortem] dissection, and while they get this in numerous cases in the large Maternity Hospitals they are not likely to get it to the same extent in household events’.^109^ In some cases, the condition of the mother might be given instead of the cause of death of the child.^110^ Yet such difficulties were not a result of lack of encouragement from the GROS, which regularly stressed the importance of accurate certification.^111^ In cases where a doctor left blank the cause of death, registrars were asked to take the matter up with the practitioner and to request more specific information, failing which the doctor should be asked to at least insert the word ‘unknown’.^112^ Not all doctors appreciated having their work questioned, however. One complained: ‘We are working under conditions of gross over-crowding and under-staffing. There is little or no time to take accurate histories and to investigate their truth’.^113^ Conversely, a junior doctor in Glasgow objected to being asked for a cause in six cases he had certified as ‘unknown’ because post-mortem examinations were said to be almost universally carried out on stillbirths within his hospital.^114^

Thus, although initially reluctant to include causation data within stillbirth registration, the Scottish Registrars General and their registrars were soon active in trying to obtain full information on causes of death, and in encouraging medical scrutiny of all cases. The GROS noted that although a ‘lazy resident’ might not always bother to ensure complete accuracy, when asked for fuller information this was usually forthcoming. The Registrar General declared himself confident that certifiers would soon realize the importance of their certificates to public health. Through such enquiries, pursued where certification was thought to be incomplete, either through so-called laziness or, more likely, simple inability to classify the death, the quality of the information was said to be continually improving.^115^

## IV

Despite some difficulties in eliciting stillbirth data, the reports and statistics of the Registrar General for Scotland quickly became an invaluable resource for those who wished to study infant and maternal mortality, and arguably stimulated much discussion on the medico-social problem of stillbirth. In his annual reports from 1939 onwards, he began to accumulate the stillbirth-related data now available to him, producing tables that indicated the distribution of stillbirths according to cause of death, geography, sex of the child, age and parity of the mother, social class of the father, and the proportion of illegitimate stillbirths. The Scottish system of stillbirth registration, through collecting causation data, also enabled investigation into the relationship between these factors and the causes of stillbirth.

Table 1 reveals the number of stillbirths registered in both England/Wales and Scotland in the years following Scottish stillbirth registration, and the rate per thousand of the total number of children born. During 1939, the total number of stillbirths registered in Scotland was 3,832, and as there were 86,899 total births registered, the stillbirth rate was 42 per 1,000 of all births. This appeared slightly higher than the corresponding rate for England and Wales (38). By 1944, the total number of stillbirths registered in Scotland was 3,221, or a rate of 32.5 per 1,000 total births. Table 1 demonstrates the steady reduction in the rate of stillbirths registered in Scotland, England, and Wales.

**Table 1:** *Total number of stillbirths registered, 1939–47*

	Rate of stillbirths per 1,000 total births, 1939–47								
	1939	1940	1941	1942	1943	1944	1945	1946	1947
Scotland	3,832	3,799	3,698	3,602	3,494	3,221	2,949	3,483	3,563
	*42*	*42*	*39.6*	*38.2*	*35.6*	*32.5*	*32.8*	*32.3*	*30.5*
England and Wales	24,309	22,731	20,876	22,383	21,262	21,306	19,333	22,915	21,795
	*38*	*36*	*35*	*33*	*30*	*28*	*28*	*27*	*24*

Source:Annual Reports of the Registrar-General for Scotland, 1939–47; Registrar-General's Statistical Review of England and Wales for 1939–47.

Other trends recorded through analysis of this data included ‘wide variations in sex ratios’,^116^ and in stillbirths to mothers based on age and parity.^117^ The stillbirth rate was found to be some 14 per cent higher for illegitimate births than for legitimate, although the relationship between the two rates was said to be affected by the lower average age of unmarried mothers.^118^ In terms of geographical distribution, individual districts revealed widely varying rates of stillbirth compared to the national average, from 91 per 1,000 births in Kinross to 25 in Bute, although, in each of these cases, the numbers were small and the rates thus not deemed statistically reliable.^119^

Analysis of cause of stillbirth engaged a significant amount of research. A comparison of these yearly findings by the Scottish Registrar General, recorded in table 2, revealed the principal difference to be the noticeable drop in the ill-defined and unknown groups. Serious efforts were made to transfer all cases to specific causes, leading to a ‘most spectacular decline’, for example, in stillbirth cases attributed to asphyxia, which numbered 341 in 1939 but only 56 by 1942.^120^ By 1944, it was noted with pride that the number of stillbirths registered with cause ‘unknown’ was less than half that recorded in 1939, having dropped from 33 per cent of recorded stillbirths to 17 per cent.^121^ By 1948, only 3 per cent were deemed inexplicable in terms of present medical knowledge, though with a further 13 per cent considered ill-defined.^122^ There was, however, a possibility that, in some cases, the underlying cause might have been known to the attending practitioner or midwife but deliberately omitted from the certificate, particularly in syphilis-related cases.^123^

**Table 2:** *Number of stillbirths recorded in Scotland under the principal causal groups, 1939–45*

	1939	1940	1941	1942	1943	1944	1945
Unknown	362	242	163	164	243	175	126
Ill-defined	901	993	592	426	431	367	366
incl. asphyxia	341	362	157	56	65	64	62
incl. prematurity	286	355	160	142	127	91	85
Disease in or accident to the mother	941	963	1,044	1,055	922	810	751
Anomalies of foetus, placenta, or cord	712	745	840	887	856	852	821
Death of foetus by injury or other cause	916	856	1,059	1,070	1,042	1,017	885
Total	3,832	3,799	3,698	3,602	3,494	3,221	2,949

Source:Annual Reports of the Registrar-General for Scotland, 1939–45.

Nonetheless, a particularly disturbing statistic noted in 1939 and regularly thereafter was that the total of antenatal and neonatal deaths was exceeded only by deaths from diseases of the heart, which were more than twice as many, and by deaths from all forms of malignant disease, which were more numerous by about 10 per cent. Antenatal and neonatal deaths were more numerous than all deaths from bronchitis and pneumonia combined, double the number of deaths from all forms of tuberculosis, and six times as many as the combined number of deaths from the four principal infectious diseases of childhood (measles, scarlet fever, whooping cough, and diphtheria). Moreover, heart disease and malignant disease were problems primarily of the elderly, while the healthy infant at this time had a life expectancy of about 60 years. It was thus evident that those deaths arising ‘mainly from conditions existing before birth’ were ‘a much more grave wastage of life than would appear merely from a recital of the numbers concerned’, constituting a social and biological problem ‘of very special importance’.^124^

Aside from the socio-medical analyses of stillbirth which the annual reports of the Registrar General had begun to provide, there was a wider movement to include stillbirth within studies relating to infant and maternal health, often utilizing GROS data. It became a common argument that, in order to get a true picture of the total loss of infant life in Scotland, it was necessary to include stillbirths within the scope of any investigation.^125^ Although throughout the Second World War it was difficult to investigate infant mortality, since there was little published information and many environmental conditions were abnormal in these years, this was precisely the period in which clinical research into stillbirth began to reap rewards. A range of studies addressed the principal causal factors of stillbirth and possible modes of reduction and prevention, including work by the Department of Health for Scotland and Dugald Baird, an Aberdeen-based obstetrician.^126^

It was quickly ascertained, for instance, that about two-thirds of stillbirths were due to antenatal conditions pre-existing at birth, and the remaining one-third to the hazards of the birth itself.^127^ Thus it was estimated that improvements in obstetrical practice and delivery management could reduce the stillbirth rate in Scotland from 42 per thousand (in 1939) to about 33 per thousand, and that extended and improved antenatal medical supervision should reduce it still further. However, there were no particular advances in medical knowledge to which the great fall in stillbirths during the Second World War could be attributed, leading to the suggestion that high foetal mortality was principally found where social conditions and the standard of living were poor.^128^ Thus, in terms of the remaining two-thirds of stillbirths, it was argued that the ‘elimination of the grosser forms of poverty’ and improvements in women's health and nutrition during growth and adolescence as well as pregnancy would be beneficial.^129^ Studies such as Baird's also revealed that the quality of a woman's environment before and soon after *her* birth influenced her reproductive efficiency and the survival chances of her children a generation later, and even those of her children's children two generations later.

An important outcome of such causation-focused stillbirth and infant mortality studies was the realization in the 1940s that infant mortality was not a monolithic subject, but should be divided into the neonatal group—the ‘hard core’ of deaths in the first month of life and before—which showed very little improvement since the beginning of the twentieth century, and the post-neonatal group of deaths occurring from the end of the first month to the end of the first year, which were deemed responsible for the steady fall in infantile mortality since 1900.^130^ As Ian Sutherland at the Institute of Social Medicine at Oxford wrote, stillbirths seemed resistant to the improving environmental conditions that had assisted the dramatic decline in post-neonatal infantile mortality after 1900.^131^ Such examinations supported an earlier contention that the causes of death immediately before and after birth were different from those in later months. Subdividing the problem made it more manageable, with post-neonatal deaths being perceived to be more easily addressed than stillbirths and neonatal mortality.^132^

Furthermore, it became accepted during this decade that ‘stillbirths and neonatal deaths present[ed] a conjoint clinical problem’.^133^ Charles McNeil, Edward Clark Professor in Child Life and Health at the University of Edinburgh, argued that it was ‘necessary to place alongside any figures of neonatal mortality the corresponding stillbirths’ since ‘the dividing line’ between the two, ‘usually so sharply drawn, [was] misleading, because the same lethal processes that destroy[ed] infant life during birth, continue[d] to operate with great power after birth’.^134^ The increasing numerical importance of *early* neonatal mortality further emphasized the rather indistinct boundary between neonatal and stillbirth mortality.^135^ Indeed, it was to encapsulate this overlap of stillbirth and early neonatal death that the concept of ‘perinatal mortality’ was proposed in 1948.^136^ The physician and epidemiologist Sigismund Peller employed this term to measure mortality from the twenty-eighth week of gestation to the end of the first week of independent life, thus treating the developmental transition from foetus to newborn child as an unbroken process.^137^ He argued that stillbirth figures were the ‘least reliable in the whole realm of vital statistics’ and that the only way to avoid mistakes was by pooling stillbirths and deaths in the first week of life, since both groups were influenced by the same factors.^138^ Thus, whereas in the nineteenth century stillbirths were grouped with miscarriages and abortions as elements of foetal loss, during the interwar years they ‘became the natural partner’ of early infant mortality, ‘separated only by a single breath’.^139^

A decade after stillbirth registration began in Scotland, the GRO in London, as a result of Scotland's apparent success in relation to stillbirth causation, began to consider implementing similar measures.^140^ The Registrar General for Scotland was asked for ‘information and guidance’ based on the Scottish experience, and in particular the limitations to and value of the statistics. His response was that the registration of stillbirth causation seemed to have been ‘reasonably well observed in practice and to have justified itself’ by generating statistically useful information.^141^

Amidst the resulting discussions of the matter in English medical and statistical circles, a variety of old arguments and potential difficulties were raised. The Central Midwives Board complained that, since deliveries by midwives were more common in England than in Scotland, too much responsibility would be placed on ‘non-experts’ to determine cause of death.^142^ It was also felt that the process generally was simpler in Scotland because of its smaller population, the high degree of hospitalization, and the concentration of experienced pathologists in the four university centres of Aberdeen, Dundee, Edinburgh, and Glasgow, to which foetuses could be sent for post-mortem examination.^143^ There was also the complaint that any such expansion in the collection of statistics placed extra work on hard-pressed doctors and midwives.^144^ It was suggested by some that better information could be obtained more cheaply by intensive local enquiries, for a decade of stillbirth registration in Scotland had produced no palpable benefits or specific actions to reduce stillbirths.^145^

The GROS responded that, while midwives did indeed certify far more stillbirths in England than in Scotland, where only 1.6 per cent of stillbirths were so certified,^146^ and while there was a larger percentage of home births in England than in Scotland, these were not impediments to death certification.^147^ About 12 per cent of stillbirths in Scotland, for example, were said to show gross malformations of the central nervous system which ‘no doctor or midwife would mistake’, and there seemed to be ‘no reason for not recording the obvious’.^148^ Enquiries were pursued where certification was thought to be incomplete, and thus the quality of the information was said to be ‘continually improving’. Moreover, GROS pressure to ascertain accurate causation had itself ‘tended to establish pathologists who [had] become quite keen on doing still births and neonatal deaths’, and were ‘considerably more expert than they were ten years ago’.^149^

During the mid-1950s, several additional events focused attention on stillbirths and the possible benefits which registering causation might have for the understanding of foetal development: the two atomic bombs dropped on Japan in 1945, with the resulting heavy loss of foetal life; possible dangers to the human stock from radio-isotopes, nuclear reactors, and bomb testing; postulated relations between medical irradiation of pregnant women and the origin of leukaemia or neoplastic disease in the unborn foetus; and the tragedy of ‘thalidomide’ babies resulting from the use of an apparently harmless drug during pregnancy.^150^ The *Report on the hazards to man of nuclear and allied radiations* contained a recommendation that more information should be collected at registration, ‘particularly in order to study causes of still-birth determined by genetic antecedents’.^151^ This appears to have created a ‘renewed and much more clearly defined pressure’ upon the English GRO, principally from the Medical Research Council, which argued that ‘the absence of any statistics on cause of stillbirths [constituted] a serious gap’.^152^ As the English Registrar General ultimately argued, neatly downplaying previous achievements in this field north of the border, while Scotland could already provide such causation data, its value was limited by the low prevalence of many specific fatal conditions in a population of only 5 million.^153^ Only by the collection of statistics on a national scale could adequate data be collected to enable environmental and social hypotheses of causation to be tested. After wide discussion of the matter,^154^ registration of the causes of stillbirth was finally introduced into England and Wales in 1960 through the Population (Statistics) Act.^155^

## V

The question of whether to register stillbirths alongside live births was a contentious issue from the very introduction of civil birth registration. As Higgs notes, such ‘deficiencies’ as lack of stillbirth registration ‘only appear so if one assumes that the *raison d’etre* of the registration system was the generation of data for demographic and medical research'.^156^ In the early period, legal considerations were more important than medical ones, and financial and administrative problems had to be overcome in any expansion of the nation's vital statistics. Nonetheless, British birth and death statistics were undoubtedly compromised by the exclusion of the significant number of stillborn children from the registration process, a circumstance that both statisticians and the medical profession frequently lamented.

By the end of the nineteenth century, comparative enquiries were serving to highlight wide discrepancies in how individual nations dealt with the subject, in addition to exposing the British lack of comprehensive statistics in this area. Notification of births aided the latter to an extent, but more comprehensive stillbirth registration was resisted for a further two decades in England, and three in Scotland. Continued resistance north of the border was only overcome on the eve of the Second World War, during a second wave of anxiety over national efficiency. It was argued that stillbirth registration would allow more accurate calculations of rates of both fertility and infant mortality, and statistical comparisons with the fuller vital statistics of England and other nations. From the medical standpoint, and more significant to the Bill's successful journey through Parliament, it was hoped that stillbirth registration would make an important contribution to understandings of infant and maternal morbidity and mortality.

Doctors and statisticians appear to have broadly welcomed the cause of stillbirth data that the Scottish registration process immediately generated. It was arguably Scotland's late move to register stillbirths that enabled it to ‘improve’ on the English system in this way, and to become a crucible for British research into the causes and prevention of stillbirth. Thus, just as the lengthy gestational period preceding compulsory registration of births and deaths in Scotland had secured advantages over English registration,^157^ a similar argument could be made in the case of stillbirth registration. Such causation data was not, however, unproblematic. Historians have noted the ‘notorious unreliability’ of infant cause-of-death information in historical populations;^158^ and such problems were even more pronounced in foetal mortality at a time when an appropriate classificatory system was entirely lacking, and when even highly qualified hospital-based doctors could struggle to diagnose the cause of any given stillbirth.

When the Registration of Still-Births (Scotland) Act came into operation on 1 January 1939, the subsequent stillbirth statistics accumulated by the Registrar General for Scotland and his analyses of that data quickly became an invaluable resource for research into infant and maternal mortality, and stimulated much discussion on the medico-social problem of stillbirth in Britain. Most notably, a new ‘perinatal’ focus quickly developed as the stillborn infant began to be seen, in partnership with the neonate, as a legitimate, and indeed significant, focus of medical and statistical interest. As a writer in the *Edinburgh Medical Journal* noted, while the ‘importance attached to the survival of the newborn infant’ had ‘varied immeasurably through the centuries’, this was a classification now receiving more consideration than ever before.^159^ The significance of foetal and neonatal deaths arguably only really became clear once stillbirths were registered, and as the cause of such deaths was increasingly scrutinized. Concurrently, Higgs notes that the General Register Office in London was beginning to discuss the broader concept of ‘reproductive wastage’—‘the loss to the community of potential human life during pregnancy and labour and in the first year of life’, which included miscarriages and abortions—and that the concept of life within statistical circles was being extended back into the pre-partum period as stillbirths were integrated into statistical and medical definitions of infant mortality.^160^

It is thus argued that the status of the foetus and newborn infant was evolving significantly in the first half of the twentieth century, due partly to the notification of births and early international statistical surveys of birth and stillbirth, but more crucially to the compulsory civil registration of stillbirth and its causes. By focusing widespread attention on the stillborn for the first time, registration arguably redefined perceptions of both foetal and infant mortality in medico-social terms. Armstrong has rightly asserted that the Registrar General's creation of a specific mortality rate for infants, and subsequent subdivision of the first year of life into smaller analysable components, not only reflects the emergence of a social awareness of infant mortality, but indeed *created* social, statistical, and medical recognition of the infant as a discrete and significant entity.^161^ The caveat should, however, be added that the Registrar General was not a conscious social engineer, but a pragmatist trying to juggle all of the agendas, practicalities, and concerns of those involved, particularly policy-makers, statisticians, doctors, and registrars, an issue with which Armstrong does not engage. Nonetheless, his argument can be extended back into the foetal period, with the introduction of stillbirth registration being both a cause and a symptom of the changing status of the stillborn child in the first half of the twentieth century.

## Footnotes

2 See, for example, Hart, ‘Beyond infant mortality’, p. 215.

3 See 16 & 17 Geo. 5 c. 48.

4 See 1 & 2 Geo. 6 c. 55.

5 See, for example, *Social History of Medicine*, 4, 3 (1991); Higgs, *Information state*; idem, *Life, death and statistics*.

6 See the special issues of *Journal of the History of Medicine and Allied Sciences*, 54, 2 (1999); *Continuity and Change*, 12, 2 (1997); and *Historical Methods*, 29, 2–3 (1996).

7 The exceptions to this are studies of a predominantly demographic nature which, recognizing the significance of foetal mortality to rates of both reproductive mortality and fertility, have attempted to estimate long-term stillbirth trends in England and Wales over the last few centuries. See, in particular, Woods, ‘Measurement of historical trends’; Wrigley, *Poverty, progress, and population*, pp. 317–50; Reid, ‘Neonatal mortality’; Hart, ‘Beyond infant mortality’; Mooney, ‘Still-births’.

8 Higgs, *Life, death and statistics*, p. 19. The main reason for the introduction of the birth and death registration system was to improve the recording of lines of descent and thus better the security of title to property. See also idem, ‘Linguistic construction’, p. 91.

9 See the extensive historical demographic work of Anderson, including *British population history*; and on infant mortality specifically, see Blaikie, ‘Infant survival chances’; Cage and Foster, ‘Overcrowding and infant mortality’; Garrett, ‘Birth spacing’; Kemmer, ‘Investigating infant mortality’.

10 For two recent exceptions, see Cameron, ‘Establishment of civil registration’; Elliot, ‘Early experiment’.

11 Armstrong, ‘Invention of infant mortality’.

12 *Scotsman*, 17 Jan. 1855. For a full account of the introduction of compulsory civil registration to Scotland, see Cameron, ‘Establishment of civil registration’.

13 *Scotsman*, 17 Jan. 1855, p. 2.

14 The examiner's duty was to scrutinize the work of the registrars, visiting each parish in his district once a year for this purpose.

15 Glaister, ‘Death certification’, p. 248. For details of the differing medico-legal status of live-born and stillborn infants under both Scots and English law, see Crowther and White, ‘Medicine, property and the law’, pp. 859–60.

16 National Archives of Scotland, Edinburgh (hereafter NAS), Papers of the General Register Office for Scotland, GRO1/1, Examiners' Notes, Perth District, 1855.

17 NAS, GRO1/1, Examiners' Notes, Lanark District, 1855.

18 NAS, GRO1/2, Examiners' Notes, Lanark District, 1856.

19 NAS, GRO1/1, Examiners' Notes, Edinburgh, Glasgow, and Leith District, 1855.

20 NAS, GRO1/2, Examiners' Notes, Perth District, 1856.

21 *23rd Annual Report, 1877*, p. xv.

22 NAS, GRO5/93, Secretary of Registrar General (for Scotland, unless otherwise stated) to A. Rooke, Registrar, Dumbarton, 30 April 1913.

23 While medical jurisprudence textbooks such as Taylor, *Medical jurisprudence* (p. 298), had long argued otherwise, it was still considered in some quarters that when the lungs sank in water, this suggested that the child had never breathed and had thus been born dead.

24 ‘Floating of the lungs’, p. 83. It was believed that the less air found in the intestinal canal, the greater the probability that the child had survived birth. See Aitchison Robertson, *Aids to forensic medicine*, pp. 46–9.

25 ‘Hydrostatic test’, p. 1567.

26 ‘Edinburgh Obstetrical Society’, p. 64.

27 While Rose (*Massacre of the innocents*, p. 176) notes that babies were ‘grossly and disproportionately vulnerable to murder’ in this period, this may have been more of a moral panic than a widespread practice.

28 Glaister, *Medical jurisprudence*, pp. 364–90.

29 That is, the child had to have reached a stage in its development that made a living birth possible, taken at this time to be completion of the seventh month of intra-uterine life, and to have been fully born. Destruction of the child *during* the parturient process—that is, as the child was in the process of being born—did not constitute an offence against the law.

30 For an historical overview of the disposal of foetal remains, and consideration of the ‘medical waste’ status of those remains, see Morgan, ‘ “Properly disposed of” ’.

31 NAS, GRO5/93, Secretary of Registrar General to R. Leach, Clerk to the Guardians, Rochdale, 8 Nov. 1912. The English Registration of Births and Deaths Act of 1874 (37 & 38 Vict. c. 88) merely required the delivery of stillborn children to a burial ground to be accompanied by a written declaration of stillbirth from either a registered medical practitioner or the person who would have registered the birth, should the child have been born alive.

32 NAS, GRO5/94, assistant secretary, Scottish Board of Health, Edinburgh, to the Registrar General, 8 May 1924.

33 NAS, GRO5/94, J. Fyfe, Secretary, to the Clerk to the Castle Douglas District Council, Sheriff Court House, Kirkcudbright, 7 Aug. 1930. In Edinburgh, it was noted that the general procedure was to obtain a medical certificate and then take the body to the cemetery for burial. See NAS, HH61/141, J. Guy, Medical Officer of Health, Public Health Department, Edinburgh, to Mr McRobbie, Department of Health for Scotland, Edinburgh, 22 Sep. 1930.

34 ‘Law and stillbirth’, p. 1674.

35 ‘Stillbirths’, p. 763.

36 ‘Registration of stillbirths’, p. 169. See also Smith, *People's health*, pp. 67–8; and Pember-Reeves, *Round about a pound*, p. 70, which discusses the ‘terrible sum’ a mother would have to produce to bury her live-born child and thus the ‘anxiety on the part of the mother to be able to prove [or, indeed, pretend] that her child was stillborn’.

37 ‘False certificates’, p. 235.

38 NAS, GRO5/1924, W. Bissett, Registrar, Gorbals Registration Office, Glasgow, to the Procurator-Fiscal, Glasgow, 8 Dec. 1927.

39 *Edinburgh Evening Dispatch*, 3 May 1928, p. 3.

40 *Glasgow Herald*, 4 May 1928, p. 6.

41 NAS, GRO5/1924, Bissett to the Registrar General, 4 May 1928.

42 See, for example, NAS, GRO5/1924, J. Dunlop, Registrar General, to the Secretary, Scottish Board of Health, 8 Dec. 1927.

43 As Morgan notes similarly in the American context: ‘The combination of high rates of infant and child death and pecuniary disincentives to burial yielded as many corpses as anatomists could want’; and ‘it is striking to note that the respectful treatment considered appropriate for adult corpses during this period often did not extend to infant or fetal remains’. See Morgan, ‘ “Properly disposed of” ’, pp. 256–7.

44 ‘Certifying of stillborn infants’.

45 ‘False certificates’, p. 235.

46 ‘Causes of infantile mortality’, p. 241.

47 ‘Infantile mortality in Scotland’, p. 200.

48 *Glasgow Herald*, 6 April 1916, p. 2.

49 ‘Early Notification of Births Bill’, p. 541.

50 It might be noted that several British cities seem to have initiated earlier efforts in this regard, particularly Huddersfield (see Marland, ‘Pioneer in infant welfare’).

51 NAS, GRO5/1924, Note by Registrar General, 13 Sept. 1913; ‘Notification of stillbirths’, p. 844.

52 *Report of the Medical Officer*, p. 43.

53 ‘Edinburgh Obstetrical Society—stillbirths registration’, p. 1540.

54 For example, the Medical Officer of Health for the County of Renfrew noted that he did ‘not propose to advise the adoption of the Act’ in areas where the population was ‘comparatively sparse, and where it [was] mainly of an agricultural or residential character’. See NAS, HH62/36, *18th Annual Report of the County of Renfrew* (1908, p. 18). The medical officer of health for the County of Kinross-shire noted ‘the great practical difficulty’ of carrying out such measures in an area of scattered and sparse population. See NAS, HH62/38, *Kinross-shire Annual Report on the Health and Sanitary Condition of the County* (1909, p. 16).

55 See 5 & 6 Geo. 5 c. 64.

56 *Scotsman*, 7 July 1915, p. 9.

57 NAS, GRO5/93, R. Rew, Honorary Foreign Secretary, Royal Statistical Society, London, to P. Macdougall, Registrar General, 1 May 1911.

58 ‘Infant mortality and stillbirths’, p. 500.

59 ‘Edinburgh Obstetrical Society’, p. 63.

60 Dudfield, ‘Still-births’, p. 1.

61 Ibid., p. 3.

62 Ballantyne, ‘Still-births' registration’, p. 9.

63 Private members' bills in 1908 and 1914 had attempted to make stillbirths registrable but were both squashed by the government on the grounds ‘that they would involve considerable public expenditure or were badly drafted or contained objectionable incidental matter’. See Rose, *Massacre of the innocents*, p. 133.

64 The subject matter of this bill was not, however, new. The *First and Second Reports from the Select Committee on Death Certification* (P.P. 1893–4, IX) made a number of recommendations that were embodied in this and previous bills of a similar nature.

65 *Registrar-General's Statistical Review 1927*, p. 128.

66 NAS, GRO5/887, J. Dunlop, Registrar General, to S. Vivian, Somerset House, London, 11 March 1926.

67 The 1926 Bill was in many respects a resurrection of the Coroners Law and Death Certification Amendment Act of 1923.

68 NAS, GRO5/887, Assistant Secretary, Scottish Board of Health, to J. Dunlop, Registrar General, 25 April 1925.

69 NAS, GRO5/887, J. Dunlop, Registrar General, to the Secretary, Scottish Board of Health, 10 April 1923.

70 NAS, GRO5/887, Note by J. Dunlop, ‘Observations on Bill to amend the law relating to the Certification of Deaths and the Disposal of the Dead, 15 Geo. V. Bill 132’, undated (*c.* Jan. 1926).

71 *Committee on Scottish Health Services Report* (P.P. 1936, XI). See also McLachlan, ed., *Improving the common weal*, pp. 75–9.

72 *Committee on Scottish Health Services Report* (P.P. 1936, XI), para. 533.

73 NAS, GRO5/95, P. Taylor, Department of Health for Scotland, to W. Murrie, Scottish Office, Whitehall, 4 April 1938.

74 See, for example, Douglas and McKinlay, *Report on maternal morbidity*.

75 NAS, GRO5/95, Taylor to Murrie, 20 April 1938.

76 NAS, GRO5/94, W. Douglas, Secretary of State, Edinburgh, ‘Department of Health for Scotland Submission on the Registration of Stillbirths’, 29 Oct. 1937.

77 That is, deaths within a year of birth per 1,000 births. NAS, GRO5/95, R. H., Department of Health for Scotland, to H. Scott, New Register House, Edinburgh, 11 Jan. 1938.

78 NAS, HH61/802, Registration of Still-Births (Scotland) Bill, undated.

79 NAS, GRO5/95, R. H., Department of Health for Scotland, to H. Scott, New Register House, 11 Jan. 1938.

80 NAS, GRO5/95, Taylor, Department of Health for Scotland, to Murrie, Scottish Office, containing ‘A Memorandum by our Medical People Regarding the Value of the Bill as a Health Measure’, 20 April 1938.

81 NAS, GRO5/94, W. Douglas, Secretary of State, Edinburgh, ‘Department of Health for Scotland Submission on the Registration of Stillbirths’, 29 Oct. 1937.

82 *H. of C. Debates*, 5th ser., vol. 332, 2 March 1938, cols. 1116–20.

83 NAS, GRO5/95, *H. of C. Parliamentary Debates*, vol. 83, Standing Committee on Scottish Bills, Registration of Still-Births (Scotland) Bill, 17 March 1938, pp. 3–33.

84 NAS, HH61/803, R. Johnson to C. Harris, Government Whips Office, 12 Downing Street, London, 5 March 1938.

85 NAS, GRO5/95, *H. of C. Parliamentary Debates*, Standing Committee on Scottish Bills, Registration of Still-Births (Scotland) Bill, 17 March 1938, p. 21.

86 Ibid, pp. 29–30.

87 *H. of L. Debates*, 5th ser., vol. 110, 5 July 1938, cols. 513–15.

88 NAS, GRO5/95, Registration of Still-births (Scotland) Bill, Explanatory Memorandum, undated.

89 NAS, GRO5/96, booklet, ‘Regulations and Instructions to Registrars, Registration of Stillbirths (Scotland) Act, 1938’. Thus stillbirth registration was conceived as it was in England, where Vivian observed that the principle adopted was that registration was not primarily a matter of public record for public use, but a contribution to statistics and an element in the machinery for the protection of infant life. The public were therefore deprived of ‘their usual right’ of getting certified copies and the stillbirth register was not publicly accessible. See NAS, GRO5/96, S. Vivian, Somerset House, London, to J. Kyd, Registrar General, 10 Nov. 1937.

90 NAS, GRO5/2013/1, Registrar General to Prof. R. J., Edinburgh, 29 March 1940.

91 NAS, GRO5/94, Registrar General to Sir S. Vivian, General Register Office (hereafter GRO), London, 5 Nov. 1937.

92 NAS, GRO5/94, Vivian, GRO, London, to J. Kyd, Registrar General, 10 Nov. 1937.

93 NAS, GRO5/94, Note of Meeting of Department of Health for Scotland, 11 Nov. 1937.

94 For further details on the greater input of Scottish medical practitioners to maternity care than their English counterparts, which appears to have been a tradition dating back as far as the seventeenth century, see Reid, ‘Scottish midwives’.

95 NAS, GRO5/95, Note by the English Registrar General, undated (*c.* March 1938).

96 NAS, GRO5/94, Department of Health for Scotland to Office of Registrar General, containing ‘Notes prepared for Major Neven-Spence for your Still Births Registration file’, 11 Jan. 1938.

97 NAS, GRO5/94, Note of Meeting of Department of Health for Scotland, 11 Nov. 1937.

98 NAS, HH61/802, Registration of Still-Births (Scotland) Bill, undated.

99 NAS, GRO5/95, Department of Health for Scotland to H. Scott, New Register House, Edinburgh, 8 April 1938. The causes given included maternal toxaemia (for example, eclampsia), deformities of the foetus (for example, hydrocephalus), and prematurity.

100 NAS, GRO5/94, Dr J. Mackintosh, Chief Medical Officer, Department of Health for Scotland, to Dr P. McKinlay, Superintendent of Statistics, Registrar General's Office, Edinburgh, 12 Nov. 1937. These registers were said to contain particulars of the mother's condition during pregnancy and confinement (that is, details as to sickness or defect during pregnancy and complications or difficulties during labour) and any obvious condition in the foetus.

101 NAS, GRO5/96, H. Scott, Office of Registrar General, to the Registrars of College, Glasgow; George Square, Edinburgh; Canongate, Edinburgh; Bellshill; Springburn, Glasgow; Govan, Glasgow; St Clement, Dundee; Northern, Aberdeen, 23 Dec. 1938.

102 *85th Annual Report, 1939*, p. lxviii.

103 Of the 3,799 stillbirths registered, the cause of death was certified in 3,620 cases by a medical practitioner and in 173 cases by a certified midwife. See *86th Annual Report, 1940*, p. xlviii.

104 See, for example, NAS, GRO5/2013/1, General Registry Office of Births, Scotland, to the Registrar, District in North Ayrshire, 20 March 1941; it was noted that about half of the stillbirths registered as having occurred in a local maternity home had been certified by midwives, and the registrar of the district was to make clear to the midwife her duty to call for medical assistance and to have the certificate signed by the doctor wherever possible.

105 NAS, GRO5/2013/1, Office of the Registrar General to GRO, London, 10 Aug. 1949.

106 Ibid.

107 NAS, GRO5/2013/1, Office of Registrar General to Department of Health for Scotland, 28 May 1945.

108 NAS, GRO5/94, Department of Health for Scotland to Office of Registrar General, containing ‘Notes prepared for Major Neven-Spence for your Still Births Registration file’, 11 Jan. 1938.

109 NAS, GRO5/96, J. Grieve, Partick Registrar, to Mr Scott, Office of Registrar General, 24 Oct. 1938.

110 See, for example, NAS, GRO5/2006/9, County Medical Officer, Dumbarton, to the Registrar General, 23 May 1940.

111 NAS, GRO5/2013/1, booklet, ‘Certificate of Still-Birth by Registered Medical Practitioner or Certified Midwife, Registration of Still-Births (Scotland) Act, 1938’, section 1(2).

112 See, for example, NAS, GRO5/2006/9, Secretary, Office of Registrar General, to the Registrar, District in Aberdeenshire, 19 April 1939.

113 NAS, GRO5/2006/5, Consultant, Rankin Memorial Hospital, Greenock, to the Secretary, GRO, Edinburgh, 1 April 1947.

114 NAS, GRO5/2013/1, Registrar, Glasgow Royal Maternity and Women's Hospital, to Superintendent of Statistics, Registrar General's Office, Edinburgh, 3 Nov. 1949.

115 TNA, PRO, MH55/1711, A. Davidson, Department of Health for Scotland, to Sir John Charles, Ministry of Health, Whitehall, 20 June 1950.

116 There were found to be 11% more male than female children among the stillborn, as against a 5% difference among the live-born. See *89th Annual Report, 1943*, p. lxiii. Sutherland, *Stillbirths*, p. 23, found a similar trend in England.

117 The number of stillbirths per thousand births was discovered to be 32 at ages under 20, and 30 at ages 20–4, but thereafter the rate increased steadily with the age of the mother to 73 at ages 40–4, and 136 at ages over 45. Similarly for parity, primiparous (first) births generally had a ‘considerably higher stillbirth rate’ than second or third births, although thereafter the rate rose with increasing numbers of previous children. Sutherland, *Stillbirths*, pp. 25–7, similarly found that in England primiparae had a higher stillbirth rate than multiparae of the same age, and that the rates for both increased after the age of 25.

118 It was calculated that the excess stillbirth rate in the unmarried mother was 45% at ages under 20, and 38% in the age group 20–5. See *85th Annual Report, 1939*, pp. lxv–vi.

119 Ibid., p. lxiv.

120 *88th Annual Report, 1942*, p. lx.

121 *90th Annual Report, 1944*, p. lxv.

122 NAS, GRO5/2013/1, Office of Registrar General to GRO, London, 10 Aug. 1949. This referred to those cases assigned a vague cause such as prematurity which was considered to be descriptive of the foetus but which did not actually explain *why* it was stillborn.

123 *86th Annual Report, 1940*, p. lviii.

124 *85th Annual Report, 1939*, p. lxxii.

125 Department of Health for Scotland, *Infant mortality*, p. 3.

126 See, for example, ibid.; Cameron and Graham, ‘Antenatal diet’; ‘Two inquiries’; Duncan, Baird, and Thomson, ‘Causes and prevention’.

127 Department of Health for Scotland, *Infant mortality*, p. 5. For the contribution of midwifery to foetal health and mortality, see Woods, ‘Lying-in and laying-out’.

128 Duncan et al., ‘Causes and prevention’, p. 183. The falling infant mortality rate and contemporary explanations of it, particularly by Dugald Baird and his colleagues in Aberdeen, have since been utilized by historians debating the living standards of the British working classes during the interwar years. See, for example, Winter, ‘Infant mortality’; Webster, ‘Healthy or hungry thirties?’; Winter, ed., *Working class*, pp. 232–55.

129 Duncan et al., ‘Causes and prevention’, p. 195.

130 Cameron and Graham, ‘Antenatal diet’; Baird, ‘Influence’.

131 Sutherland, ‘Stillbirth-rate’.

132 Department of Health for Scotland, *Infant mortality*, p. 35.

133 McNeil, ‘Discussion on stillbirth’, p. 63.

134 Ibid., p. 61.

135 Armstrong, ‘Invention of infant mortality’, p. 216.

136 Peller, ‘Mortality, past and future’.

137 Hart, ‘Beyond infant mortality’, p. 220.

138 Peller, ‘Mortality, past and future’, p. 413.

139 Armstrong, ‘Invention of infant mortality’, p. 215.

140 NAS, GRO5/2013/1, GRO, London, to Office of Registrar General, Edinburgh, 3 Aug. 1949. At this point, the Ministry of Health pursued a policy of encouraging local authorities to investigate the causes of stillbirth in their own areas. The Public Health Department of London County Council, for example, sent out a circular requesting that doctors in that sub-district inform either the Coroner's Office or local police station of any stillbirth or neonatal death in their area, and ‘obtain all possible information, for research purposes, regarding the cause of death and stillbirth of all children under 28 days’ (TNA, PRO, RG48/2177, B. Stockbridge, Registrar, Upper Holloway Sub-District, to the English Registrar General, London, 15 Feb. 1950).

141 NAS, GRO5/2013/1, Office of Registrar General to GRO, London, 10 Aug. 1949.

142 TNA, PRO, MH55/1711, G. North, GRO, Somerset House, London, to Sir John Charles, Ministry of Health, 30 Aug. 1952.

143 TNA, PRO, MH55/1711, Dorothy Taylor to C. M. O., 8 Dec. 1950.

144 TNA, PRO, MH55/1711, Arnold Walker, Central Midwives Board, Westminster, to Sir John Charles, Ministry of Health, Whitehall, 22 April 1950.

145 TNA, PRO, MH55/1711, G. North, GRO, London, to Sir J. Charles, Ministry of Health, 30 Aug. 1952.

146 TNA, PRO, MH55/1711, Note by C. Douglas, Department of Health for Scotland, ‘Causes of Stillbirth’, 3 May 1950.

147 TNA, PRO, MH55/1711, A. Davidson, Department of Health for Scotland, to J. Charles, Ministry of Health, Whitehall, 20 June 1950.

148 Ibid.

149 TNA, PRO, MH55/1711, Dr C. Douglas, Department of Health for Scotland, to Dr D. Taylor, Ministry of Health, Whitehall, 27 Nov. 1950.

150 *Registrar-General's Statistical Review 1961*, p. 189.

151 TNA, PRO, MH55/1711, North, GRO, London, to Charles, Ministry of Health, 22 Jan. 1958.

152 TNA, PRO, MH55/1711, North, GRO, London, to Charles, Ministry of Health, 22 Jan. 1958; G. Godber, Ministry of Health, Whitehall, to W. Sheldon, Harley Street, London, 29 Jan. 1958.

153 TNA, PRO, MH55/1711, Note from Registrar-General, undated (*c.* Jan. 1958).

154 See, in particular, TNA, PRO, files MH55/1711, RG26/162-3, and RG26/440.

155 See *Registrar-General's Statistical Review 1961*, p. 189; 8 & 9 Eliz. 2 c. 32.

156 Higgs, *Life, death and statistics*, p. 18.

157 For the benefits to the Scottish system of the 18-year delay between compulsory civil registration in England and Scotland, see Cameron, ‘Establishment of civil registration’.

158 See, for example, Reid, ‘Neonatal mortality’, p. 225.

159 Ellis, ‘Newborn’, p. 321.

160 Higgs, ‘Linguistic construction’, p. 92.

161 Armstrong, ‘Invention of infant mortality’, p. 212.

## Official publications

*Committee on Scottish Health Services Report* (P.P. 1936, XI).

*First and Second Reports from the Select Committee on Death Certification* (P.P. 1893–4, IX).

*Report of the Medical Officer of Health of the City of Glasgow* (1908).

*23rd Annual Report of the Registrar-General of Births, Deaths and Marriages in Scotland, 1877* (1881).

*85th Annual Report of the Registrar-General for Scotland, 1939* (1944).

*86th Annual Report of the Registrar-General for Scotland, 1940* (1944).

*87th Annual Report of the Registrar-General for Scotland, 1941* (1944).

*88th Annual Report of the Registrar-General for Scotland, 1942* (1945).

*89th Annual Report of the Registrar-General for Scotland, 1943* (1946).

*90th Annual Report of the Registrar-General for Scotland, 1944* (1946).

*91st Annual Report of the Registrar-General for Scotland, 1945* (1948).

*92nd Annual Report of the Registrar-General for Scotland, 1946* (1949).

*93rd Annual Report of the Registrar-General for Scotland, 1947* (1950).

*The Registrar-General's Statistical Review of England and Wales for 1927* (1929).

*The Registrar-General's Statistical Review of England and Wales for 1939* (1944).

*The Registrar-General's Statistical Review of England and Wales for 1940* (1944).

*The Registrar-General's Statistical Review of England and Wales for 1941* (1945).

*The Registrar-General's Statistical Review of England and Wales for 1942* (1946).

*The Registrar-General's Statistical Review of England and Wales for 1943* (1948).

*The Registrar-General's Statistical Review of England and Wales for 1944* (1949).

*The Registrar-General's Statistical Review of England and Wales for 1945* (1947).

*The Registrar-General's Statistical Review of England and Wales for 1946* (1948).

*The Registrar-General's Statistical Review of England and Wales for 1947* (1949).

*The Registrar-General's Statistical Review of England and Wales for 1961* (1963).
